# Tissue Specific DNA Methylation in Normal Human Breast Epithelium and in Breast Cancer

**DOI:** 10.1371/journal.pone.0091805

**Published:** 2014-03-20

**Authors:** Ayelet Avraham, Sean Soonweng Cho, Ronit Uhlmann, Mia Leonov Polak, Judith Sandbank, Tami Karni, Itzhak Pappo, Ruvit Halperin, Zvi Vaknin, Avishay Sella, Saraswati Sukumar, Ella Evron

**Affiliations:** 1 Department of Oncology, Assaf Harofeh Medical Center, Affiliated with Tel Aviv University, Zerifin, Israel; 2 Department of Oncology, Johns Hopkins University, School of Medicine, Baltimore, Maryland, United States of America; 3 Department of Pathology, Assaf Harofeh Medical Center, Affiliated with Tel Aviv University, Zerifin, Israel; 4 Department of Surgery, Assaf Harofeh Medical Center, Affiliated with Tel Aviv University, Zerifin, Israel; 5 Department of Genecology, Assaf Harofeh Medical Center, Affiliated with Tel Aviv University, Zerifin, Israel; Institut national de la santé et de la recherche médicale, France

## Abstract

Cancer is a heterogeneous and tissue-specific disease. Thus, the tissue of origin reflects on the natural history of the disease and dictates the therapeutic approach. It is suggested that tissue differentiation, mediated mostly by epigenetic modifications, could guide tissue-specific susceptibility and protective mechanisms against cancer. Here we studied breast specific methylation in purified normal epithelium and its reflection in breast cancers. We established genome wide methylation profiles of various normal epithelial tissues and identified 110 genes that were differentially methylated in normal breast epithelium. A number of these genes also showed methylation alterations in breast cancers. We elaborated on one of them, TRIM29 (ATDC), and showed that its promoter was hypo-methylated in normal breast epithelium and heavily methylated in other normal epithelial tissues. Moreover, in breast carcinomas methylation increased and expression decreased whereas the reverse was noted for multiple other carcinomas. Interestingly, TRIM29 regulation in breast tumors clustered according to the PAM50 classification. Thus, it was repressed in the estrogen receptor positive tumors, particularly in the more proliferative luminal B subtype. This goes in line with previous reports indicating tumor suppressive activity of TRIM29 in estrogen receptor positive luminal breast cells in contrast to oncogenic function in pancreatic and lung cancers. Overall, these findings emphasize the linkage between breast specific epigenetic regulation and tissue specificity of cancer.

## Introduction

Whereas the DNA sequence is almost identical in every cell of the body, varying epigenetic modules, including DNA methylation, chromatin modifications and regulatory RNAs, determine differentiation and tissue specificity [1], [2]. During tumorigenesis, numerous genetic and epigenetic alterations, as well as changes in the microenvironment, generate the cancerous phenotype [3], [4]. Yet, the tissue of origin can usually be recognized and often dictates the clinical behavior of the disease as well as response to therapy. As recently shown [5], [6], regions in the genome that show frequent DNA methylation variation among normal tissues are also differentially methylated and hyper variable in cancers, and therefore relate epigenetic regulation of differentiation to carcinogenesis. Accordingly, the identification of tissue-specific epigenetic regulation may throw light on cancer protective mechanisms and disclose new players in carcinogenesis. As most human solid tumors are of epithelial origin, we established genome wide methylation profiles of various normal epithelial tissues and identified a set of genes that had a distinct methylation pattern in the breast as compared to the other tissues. Functional classification of these genes revealed a subgroup of DNA binding proteins and transcription regulators that have special relevance to breast cancer. We then focused on one of these genes for detailed analysis, and selected TRIpartite Motif–containing 29 (TRIM29), also called the ataxia telangiectasia group D–complementing (ATDC), that belongs to the TRIM protein family [7]. We chose this gene because it was previously shown that it had opposing functions in cancers depending on the specific tissue milieu. Thus, over-expression of TRIM29 was observed in many human cancers [7] including pancreatic cancer, where it enhanced tumor growth through stabilization of beta catenin [8]. Additionally, TRIM29 promoted cell proliferation through interaction and suppression of p53 transcriptional activity [9]. In contrast, reduced expression of TRIM29 was noted in breast and prostate cancers [10], [11] and evidence for growth inhibition and tumor suppression was demonstrated in breast non-malignant and malignant cell lines [12]. Overall, these studies showed that TRIM29 could function either as an oncogene or as a tumor suppressor gene in various epithelial tissues. Therefore, we considered it a prominent candidate for elaborating on the relevance of breast-specific epigenetic regulation to carcinogenesis.

## Results

### Genome wide methylation analysis of normal epithelial tissues

**Figure 1:**
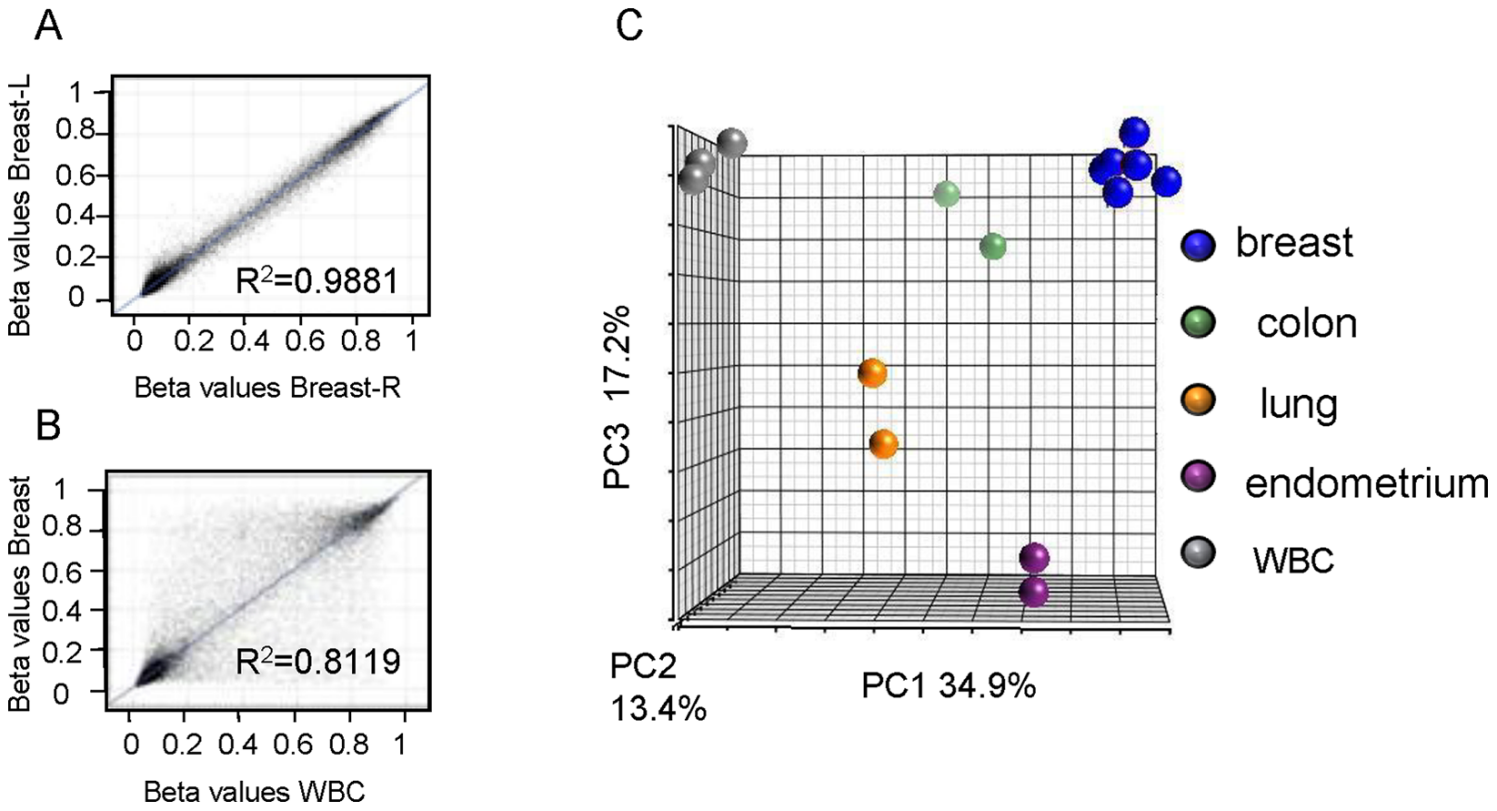
Genome-wide methylation analysis of various normal human epithelial tissues. Pearson correlation coefficient was calculated (**A**) between the right and the left breast and (**B**) between breast epithelium and WBC of the same woman. (**C**) Principal Component Analysis (PCA) grouped 15 tissue samples by their global methylation resemblance into 5 clusters that corresponded with the origin of the tissues.

To recognize breast specific methylation patterns at gene promoter sites we established genome-wide methylation profiles of normal human epithelial tissues by Illumina-Infinium HumanMethylation27 arrays. Included tissues were breast (n = 6), colon (n = 2), lung (n = 2) and endometrium (n = 2), as well as white blood cells (WBC, n = 3). To study the epithelial component specifically and reduce tissue heterogeneity, special care was taken to select the epithelial layers from the surgical specimens and to enrich the breast tissues for epithelial cells (organoids) [13]. To evaluate the consistency of the array data we calculated the Pearson correlation coefficient for similar and diverse tissues from the same individual. Strong correlation was found between the right and left breasts of the same woman (**Fig. 1A**) while weaker correlation was shown when breast epithelium and WBC of that woman were compared (**Fig. 1B**). In addition, Principal Component Analysis (PCA) grouped the samples by their global methylation resemblance into 5 clusters that corresponded with the origin of the tissues (**Fig. 1C**). This demonstrated that normal epithelial tissues from different organs had unique methylation profiles.

**Figure 2:**
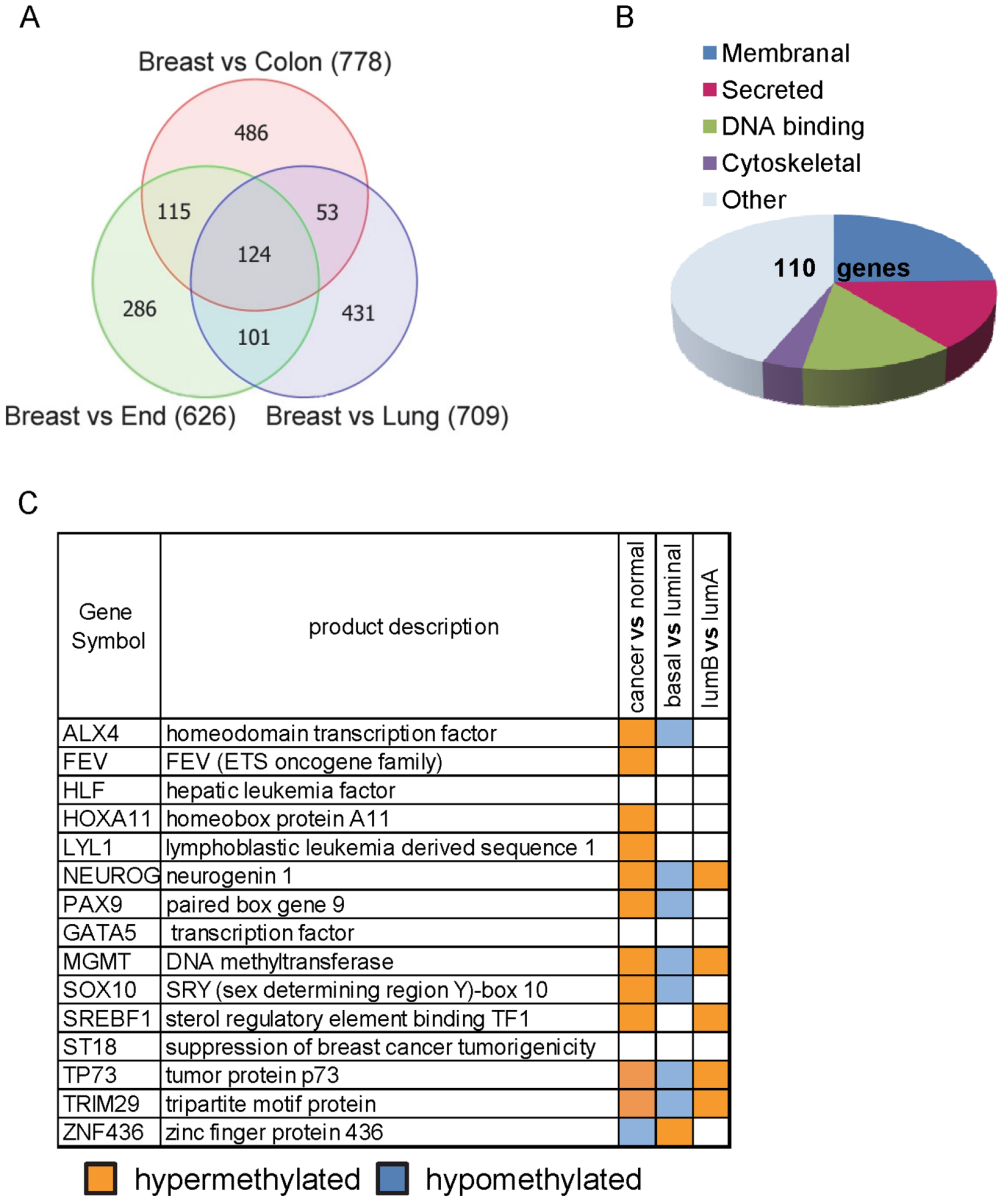
Differentially methylated promoter regions in normal breast epithelium. (**A**) Venn diagram showing differential methylation loci in breast epithelium (organoids, n = 6) as compared to colon (n = 2), lung (n = 2) and endometrial (End) epithelial tissues (n = 2). About 700 loci (number indicated in brackets) were differentially methylated in breast epithelium when compared to either of the colon, lung or endometrial epithelium. 124 loci had unique methylation patterns in the breast as compared to the other epithelial tissues. Selection of differentially methylated loci was based on a β-value difference >0.2 and a P-value <0.05, after false discovery rate correction, using analysis of variance (ANOVA). (**B**) Functional classification of 110 genes corresponding to the 124 differentially methylated probes (DAVID Bioinformatics Resources 6.7, NIAID). (**C**) Selected sub-group of breast specific methylated genes encoding for DNA binding proteins. TCGA analysis (BRCA methylation Illumina 450 dataset, p<0.0001 calculated using Bonferroni corrected t-test) revealed methylation alterations in breast cancers (n = 497) as compared to normal breast tissues (n = 97), in basal (n = 31) versus luminal (n = 144) breast tumors and in luminal-A (n = 99) versus luminal-B (n = 45) tumors. The genomic region analyzed for each gene corresponds with the Illumina probes that were found differentially methylated in breast as specified in Table S3.

To identify genes that are differentially methylated in mammary epithelium we compared the methylation profile of the breast organoids to the profiles of the other tissues. Interestingly, 2,429 loci were differentially methylated in normal breast epithelium as compared to WBC (not shown) and about 700 loci were differentially methylated in mammary epithelium when compared to colon, lung or endometrial epithelium. Furthermore, 124 loci that corresponded to 110 genes had unique methylation pattern in the breast (n = 6) as compared to all other epithelial tissues tested (n = 6) (**Fig. 2A** **and Table S1**). Methylation folds differences ranged from −1.5 (hypomethylated) to +1.5 (hypermethylated) and were consistent within the individual comparisons (**Table S1 and Fig. S1**). Functional classification of the 110 genes by DAVID [14] indicated several categories (**Fig. 2B** **and Table S2A**). The most significant groups were: secreted and extracellular proteins (18 genes, enrichment score 2.2), calcium homeostasis (4 genes, enrichment score 1.4) and transcription regulation (15 genes, enrichment score 1.3). We further elaborated on the DNA binding and transcription regulators for their notable relevance to cancer. Thus, a literature search for members of this group including ALX4, GATA5, MGMT, NEUROG1, SOX10, SREBP1, ST18, TRIM29 and TP73 revealed their evident roles in differentiation, cancer and epigenetic regulation (described in details in **Table S2B**). Moreover, analysis of The Cancer Genome Atlas (TCGA) data base [15] revealed methylation alterations for most of these gene loci in breast cancers (n = 497) as compared to normal breast tissues (n = 97), (**Fig. 2C**). In addition, for some of these gene loci methylation also varied among breast cancer subtypes: basal tumors (n = 31) versus luminal (n = 144), and luminal-A (n = 99) versus luminal-B (n = 45), (**Fig. 2C**). Furthermore, methylation at these loci also varied in other tumor types as compared to the respective normal tissues (lung, colon and endometrium, TCGA analysis, **Table S3**). This defined these gene loci as differentially methylated regions (DMR), in line with previous findings in colon [6]. We then focused on one of these DNA binding genes for detailed analysis. We selected TRIM29 because of previous findings that revealed opposing functions of this gene in various cancers. Thus, it was tumor suppressing in breast cancers [12] but oncogenic in pancreatic and lung cancers [8], [16]. These findings pointed to TRIM29 as a potential candidate to address the linkage between epigenetic regulation of this gene in normal tissues and its role in the respective carcinomas.

### Differential epigenetic regulation of TRIM29 in normal human breast epithelium

**Figure 3:**
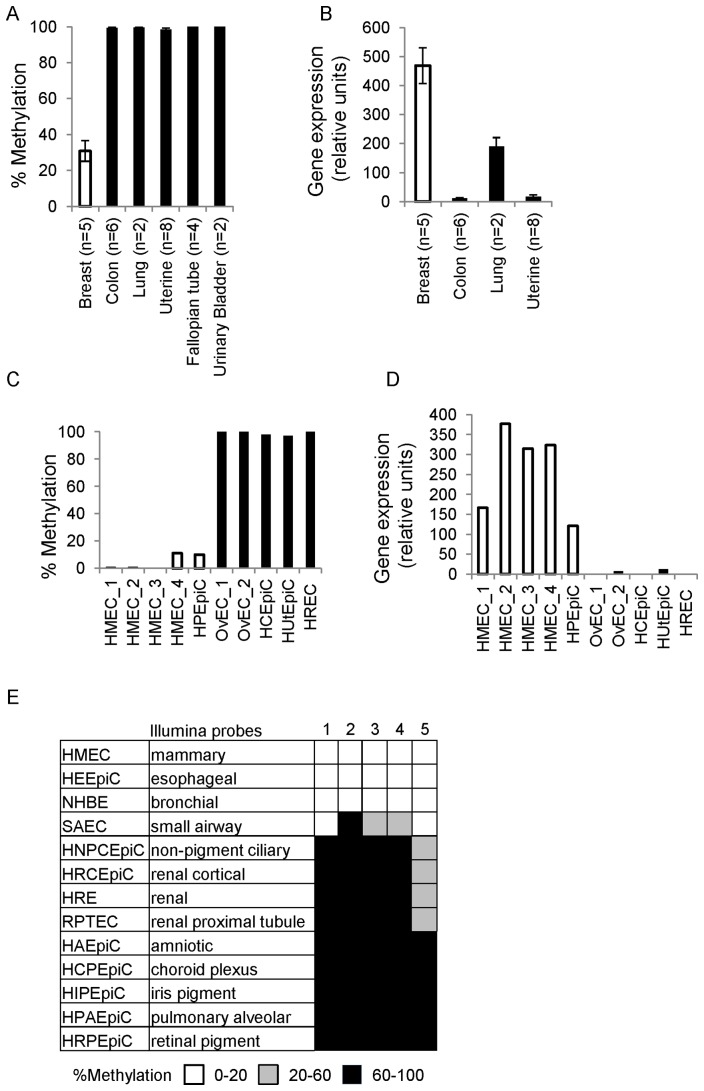
TRIM29 promoter methylation and gene expression varies in normal epithelial tissues. (**A&C**) Q-MSP at TRIM29 promoter in (**A**) various human normal tissues enriched for epithelium and (**C**) purified human normal epithelial cells. The same tissue samples studied by the Illumina methylation array were included in (A). Q-MSP amplicon overlapped with the sequence of Illumina probe #cg13625403 used for array analysis (**Table S4**). (**B&D**) The same samples were used for gene expression analysis by qRT-PCR that showed inverse correlation with promoter methylation (RNA was not available for fallopian tube and urinary bladder). HMEC- human mammary epithelial cells, HPEpiC-prostate, OvEC-ovarian, HCEpiC- colon, HUtEpiC-uterine, HREC-renal. (**E**) Data from Illumina Infinium methylation450K array obtained from ENCODE showed similar pattern of TRIM29 promoter methylation in additional purified human normal epithelial cells. Methylation at 5 Illumina probes is shown: 1.cg20655548 2.cg12201660 3.cg17971587 4.cg13285004 5.cg13625403 (**Table S4B**). Each row refers to one individual.

TRIM29 harbors a CpG island at the transcription start site of the gene, which was represented by probe #cg13625403 in the Illumina-Infinium HumanMethylation27 array (**Table S4A**). For this probe, the array results revealed about 50% methylation in breast organoids as compared to full methylation in the other epithelial tissues tested (**Table S4C**). Quantitative Methylation Specific PCR (Q-MSP) at this site for the same samples and for additional normal epithelial tissues confirmed the array results and revealed 100% promoter methylation in colon (n = 6), lung (n = 2), endometrial (n = 8), fallopian tube (n = 4) and urinary bladder (n = 2) as compared to only 30% in breast organoids (n = 5) (**Fig. 3A**). The expression of TRIM29 inversely correlated with the promoter methylation and was higher in normal breast organoids than in the other tissues (**Fig. 3B**). To eliminate possible bias due to tissue heterogeneity, we further purified and tested various normal primary epithelial cells. By Q-MSP, the promoter of TRIM29 was unmethylated in purified mammary epithelial cells (n = 4), (**Fig. 3C**). In contrast, the promoter was fully methylated in purified ovarian (n = 2), colonic (n = 1), uterine (n = 1) and renal (n = 1) epithelial cells. For prostate epithelial cells (n = 1) 15% methylation was noted (**Fig. 3C**). In these purified cells as well, methylation correlated with repression of the gene (**Fig. 3D**). Analysis of Illumina Infinium methylation450K array data from ENCODE/HAIB [17] confirmed our findings in additional epithelial cells (n = 1 for each cell type, **Fig. 3E**) indicating a differential mode of TRIM29 methylation and expression in normal mammary epithelium as compared to other epithelial tissues.

### Bidirectional “switch” in TRIM29 regulation from normal epithelium to the respective carcinomas

**Figure 4:**
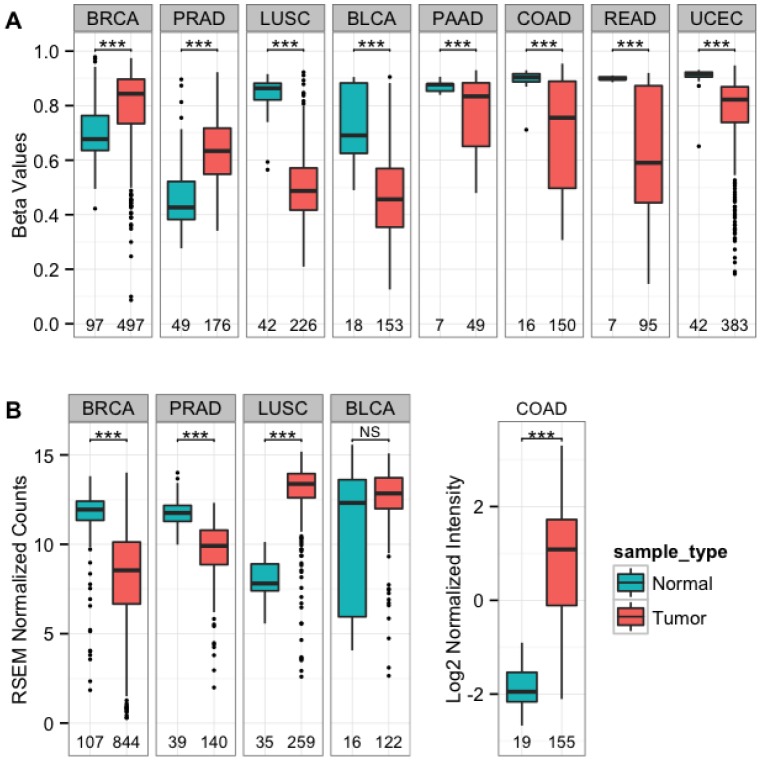
Bidirectional “switch” in TRIM29 regulation from normal epithelium to the respective carcinomas. TCGA data analysis: (**A**) TRIM29 promoter methylation (average of 5 Illumina probes, **Table S4B**) in normal and in cancer tissues. Partial methylation was noted in breast and prostate normal tissues that increased in the respective cancers. In contrast, for the other tissue-types, methylation was high in the normal tissues and decreased in the respective carcinomas. (**B**) For breast and prostate cancers, increase in promoter methylation was associated with decrease in gene expression whereas the opposite was noted for the other tissues. BRCA = breast carcinoma, PRAD = prostate adenocarcinoma, LUSC = lung squamous cell carcinoma, BLCA = bladder carcinoma, PAAD = pancreatic adenocarcinoma, COAD = colon adenocarcinoma, READ = rectal adenocarcinoma UCEC = uterine-cervix carcinoma. Number of samples indicated at the bottom, ***p<0.0001, NS – not significant, P-values between groups were calculated using Welch's corrected t-test.

To elaborate on the alterations in TRIM29 regulation between various normal tissues and the corresponding carcinomas, we analyzed promoter methylation and gene expression in TCGA data base. In line with our findings, methylation of TRIM29 in normal breast and prostate was significantly lower than in other normal tissues (**Table S5**). As can be noticed, TRIM29 methylation in crude normal breast tissues was higher (∼70%, **Fig. 4A**) than that in organoids (∼30%, **Fig. 3A**) and purified HMEC (0–10%, **Fig. 3C**). These differences originated from the fraction of non-epithelial cells in the crude samples. Accordingly, we measured 90–100% methylation in WBC (n = 6) and in primary fibroblasts (n = 3).

Interestingly, methylation increased in the breast and prostate tumors (**Fig. 4A**, sample numbers at the bottom) whereas the reverse was noted for various other epithelial tissues including lung, urinary-bladder, pancreas, colon, renal and uterine-cervix, where the promoter was heavily methylated in the normal tissues and methylation decreased in the tumors (**Fig. 4A**, sample numbers at the bottom). Evidently, the expression of the gene in the normal and cancerous tissues was altered in accordance with the promoter methylation (**Fig. 4B**). These findings indicate that epigenetic regulation of TRIM29 switches in both directions upon transformation from normal tissues to the respective carcinomas.

### TRIM29 methylation and expression in breast tumors segregate in accordance with the PAM50 subtype classification

**Figure 5:**
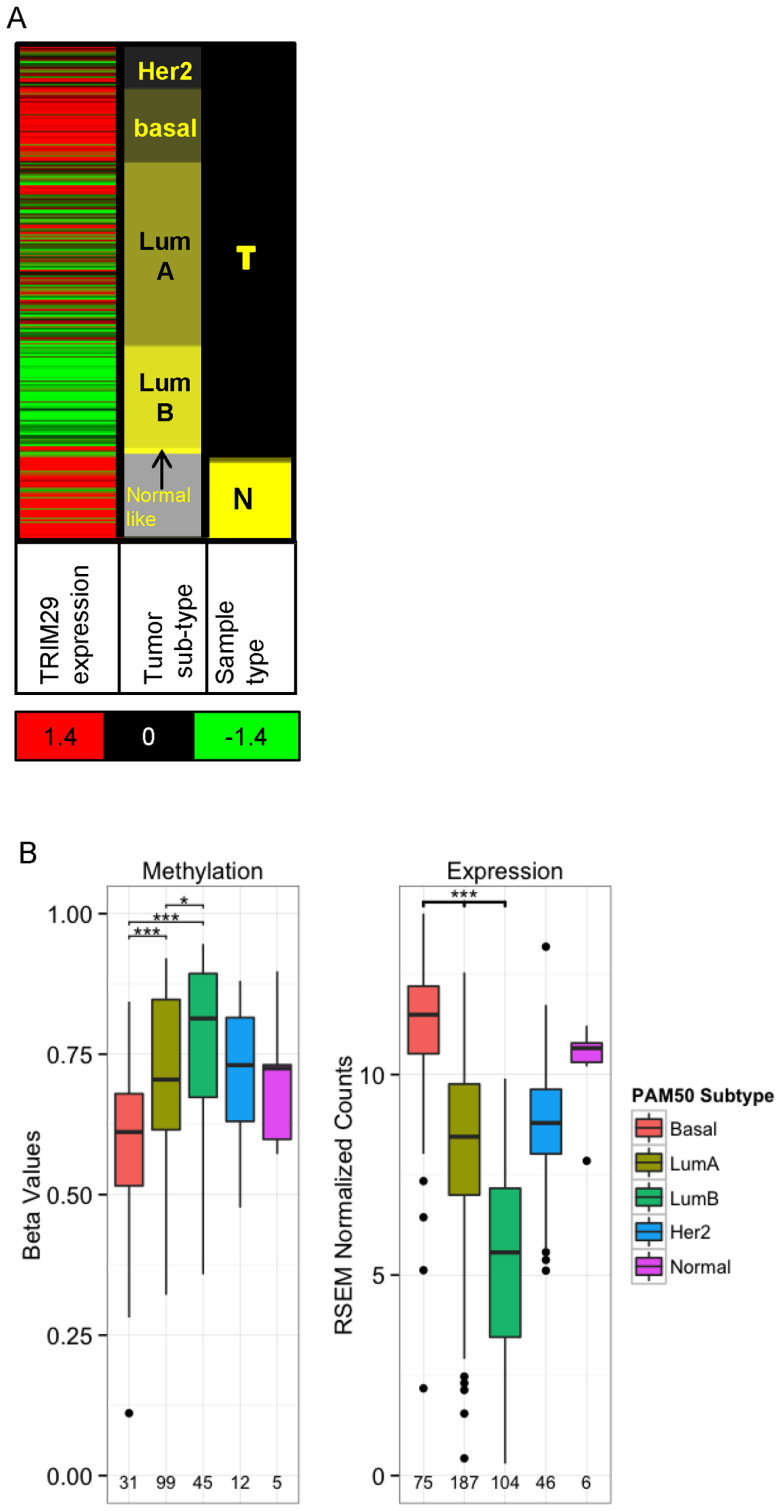
TRIM29 gene expression and promoter methylation varies in breast cancer subtypes. Analysis of TCGA data base shows (**A**) variable gene expression of TRIM29 in normal breast tissues (n = 97) and in breast tumor subtypes defined by the PAM50 classification: basal (n = 75), luminal A (n = 187), luminal B (n = 104), HER2 (n = 46), normal-like (n = 6). Heat map scale ranges from red (high) to green (low). (**B**) Variable methylation at the promoter of TRIM29 (Illumina probe #cg13625403) in breast tumor subtypes. Expression inversely correlates with promoter methylation. Data was selected from TCGA data base as described in methods. Number of samples indicated at the bottom, ***p<0.001, *p<0.01, P-values between groups were calculated using Welch's corrected t-test.

Breast cancer is a heterogeneous disease that varies by tumor genotype, phenotype and clinical parameters. Gene expression profiling established an “intrinsic gene set” [18] that defined 5 sub-types of breast tumors with distinct biological properties and clinical behavior: luminal A, luminal B, HER2-enriched, basal-like and normal-like tumors [19]. A narrowed list of 50 genes, called the PAM50 gene set, has been validated for classification of breast tumors to the intrinsic subtypes with a significant prognostic and predictive value [20]. We looked at TRIM29 methylation and expression in the different subtypes of breast cancers within the TCGA data base. Robust expression was noted in the estrogen receptor negative, basal-like group (n = 75), which mirrored normal breast tissues (n = 97). However, in the luminal, estrogen positive sub-types, expression was reduced and this was particularly evident in the luminal B subgroup (**Fig. 5A**, LumA n = 187, LumB N = 104). These differences were highly statistically significant (P<0.001, t-test, **Fig. 5B**, right panel). Of note, methylation at the TRIM29 promoter also segregated by the PAM50 subtypes (**Fig. 5B**, left panel), in inverse correlation with gene expression. These findings indicated linkage between TRIM29 regulation and intrinsic properties of breast tumors, as defined by the PAM50 classification.

## Discussion

Tissue differentiation that dictates the unique structure and function of normal tissues is regulated by epigenetic mechanisms [21]. Moreover, it is suggested that tissue-specific regulated genes may affect the susceptibility of the tissue to cancer and characterize the specific phenotype and clinical behavior of the cancer. Accordingly, the recognition of tissue-specific epigenetic patterns may disclose new players in carcinogenesis and cancer protective mechanisms. Because breast carcinomas, as well as most other human solid tumors, derive from epithelial cells, these were the subject of this study. Here we compared genome wide methylation profiles of breast epithelium to various other epithelial tissues. We identified 110 genes that were differentially methylated in the breast and focused on the subgroup of DNA binding proteins because of their apparent relevance to cancer. Analysis of TCGA data base revealed methylation alterations at most of these genes in breast cancers as compared to normal breast. Methylation also varied among breast cancer subtypes and mainly distinguished basal from luminal cancers. This is compliant with Irizarry et al. who showed that differentially methylated regions in colon cancer compared to normal colon overlapped with sites that were variably methylated among normal tissues (colon, brain, liver and spleen) and distinguished them from each other [6]. Subsequently it was shown that these variably methylated regions also distinguished cancer from normal for other tumor types (lung, breast, thyroid, and kidney) [5]. The authors therefore proposed that epigenetic alterations affecting tissue-specific differentiation are the predominant mechanism by which epigenetic changes cause cancer [6]. In accordance, a number of the breast-specific methylated transcription factors identified in this work were previously linked to breast differentiation and carcinogenesis. Thus, loss of **ALX4** caused defective mouse mammary epithelial morphogenesis and **ALX4** expression was reduced in breast cancers [22], **GATA5** was associated with hPR-B expression in mammary cells and might contribute to breast cancer risk [23], **MGMT** methylation differed between DCIS and invasive breast cancers [24], **SOX10** expression was linked to differentiation and transformation of myoepithelial/basal breast cells [25], **ST18** is a tumor suppressor that was hypermethylated and silenced in breast cancers [26] and **TP73** down-regulation led to epithelial to mesenchymal transition and marked proliferation and migration of mammary epithelial cells [27]. Of special interest, for **TRIM29** it was previously reported that it could either function as a tumor suppressor in luminal ER positive breast cells [12] or as an oncogene in pancreatic, lung and various other cancers [8], [16] [7]. Moreover, high expression of TRIM29 correlated with aggressive parameters and poor prognosis in gastric and lung cancers [16], [28]. Therefore, we further elaborated on the epigenetic regulation of this gene in normal and cancer tissues. Both methylation arrays and Q-MSP analysis showed that TRIM29 was unmethylated in normal breast epithelium and heavily methylated in various other normal epithelial cells and tissues. As expected, promoter methylation correlated with gene repression. Interestingly, TRIM29 was differentially regulated in the respective carcinomas as well, but in the reverse direction. Thus, the promoter was hyper-methylated and the gene repressed in breast and prostate carcinomas while it was de-methylated and re-expressed in the other types of carcinomas. Notably, while hypermethylation and silencing of tumor suppressor genes in cancer is commonly described, de-methylation and re-expression of oncogenes has rarely been shown [29]. It suggests that TRIM29 has tissue specific multifaceted functions upon transformation to cancer.

Looking more closely at the breast cancers, we noted that TRIM29 was methylated and suppressed mostly in the ER-positive tumors. These tumors are typically subdivided into luminal-A, which have low proliferative potential and predict good long term clinical outcome, and luminal-B that are more proliferative and indicate aggressive clinical behavior and shorter survival [30]. Notably, TRIM29 suppression was more evident in the luminal B subtype than in the luminal A. Indeed, it was previously suggested that TRIM29 might modulate the growth effects of 17β-estradiol on ER-positive mammary epithelial cells and function as a tumor suppressor in these cells. Moreover, low expression of the gene in ER-positive breast cancers was associated with worse prognosis in premenopausal but not in postmenopausal patients [12]. Taken together, these findings suggest that TRIM29 is involved in the estrogen receptor pathway and may have tumor suppressor activity in luminal mammary cells.

In summary, in this study we identified genes that are differentially methylated in normal breast epithelium, change their methylation patterns in breast cancers and may be involved in the evolution of cancer. This contributes to our understanding of the linkage between epigenetic programming, differentiation and cancer and could reveal new players in breast carcinogenesis.

## Materials and Methods

### Ethics statement

The use of human tissues in this study was approved by the Assaf Harofeh Medical Center Institutional Review Board, authorized by the Ministry of Health (Institutional Helsinki committee, written approval #35/10). All surgical specimens or blood samples were obtained after explanation to the patient or healthy donor and after his/her written and signed informed consent.

### Tissue samples, primary cells and cell lines

All samples mentioned below were collected and handled in compliance with the IRB constrains. Fresh normal breast tissues (n = 6) were obtained from reduction mammoplasties and prophylactic mastectomies. Epithelial layers of various normal human tissues were obtained from surgical specimens of operated patients (colon n = 2, lung n = 2, endometrium n = 2). White blood cells were contributed by healthy donors (n = 3). HMEC_2–4 (n = 3) and human epithelial cells of ovary (n = 2), colon (n = 1) and uterine (n = 1) were purified from primary tissues as described below. Human mammary epithelial cells (HMEC_1) were ordered from PromoCell, and human prostate and renal epithelial cells from ScienceCell.

### Enrichment for epithelial cells and preparation of primary cell cultures

Breast organoids (epithelial cells) were separated, enriched and cultured as previously described [31]. Ovarian epithelial cells were isolated and cultured according to published protocols [32]. We used a similar method to culture colon and endometrial primary epithelial cells. Cells of commercial origins were cultured according to the supplier's protocols. Cell extracts for methylation and expression analysis were prepared at exponential growth phase (passage 2–4, less than 14 days in culture).

### Genome-wide methylation analysis

Illumina-Infinium Human Methylation27 array was used for analysis of 24 samples including breast, colon, lung and endometrial epithelial tissues as well as white blood cells. Human sperm DNA and DNA treated with SssI CpG methyltransferase (NEB) were used as under-methylated and fully methylated DNA controls, respectively. Methylation scores (β values, 0–1) were generated for each of the 27,578 CpG loci (14,000 genes) on the array, based on the ratio of methylated to methylated+unmethylated signal-outputs. Partek® Genomics SuiteTM, version 6.5 was used for principal component analysis (PCA). β-average was calculated for each group of samples of the same origin, and selection of the most differentially methylated CpG loci was based on a β-average difference >0.2 and a P-value <0.05, after false discovery rate (FDR) correction, using analysis of variance (ANOVA). This part of the work was carried out at the genomics core service facility, the Rappaport research institute, Technion, Haifa, Israel.

### Data access

The data discussed in this publication have been deposited in NCBI's Gene Expression Omnibus [33] and are accessible through GEO Series accession number GSE54025 (http://www.ncbi.nlm.nih.gov/geo/query/acc.cgi?acc=GSE54025).

### Quantitative Methylation-Specific PCR (Q-MSP)

Genomic DNA was purified by standard methods using TNES-ProteinaseK treatment, phenol-chloroform extraction and ethanol precipitation. Sodium bisulfite treatment, PCR conditions and calculations were previously described [13]. Primer sequences are available in **Table S4D**.

### Gene expression analysis

RNA was isolated using the SV Total RNA isolation kit (Promega). Verso cDNA synthesis kit and Absolute Blue SYBR Green ROX mix (Thermo Fisher Scientific Inc.) were used for RT-PCR. Real-time PCR (Rotor Gene 3000, Corbett) included amplification cycles of 5 sec at 95°c followed by 30 sec at 60°c. Primer sequences are available in **Table S4E**.

### TCGA data analysis

Methylation data for the different cancer types were downloaded from The Cancer Genome Atlas (TCGA) portal (https://tcga-data.nci.nih.gov/tcga/) [15]. Level 3 data and clinical annotation tables were downloaded for all samples analyzed with the Illumina Human Methylation 450 k Array. Expression data of the different cancers were downloaded from the UCSC Cancer Genomics Browser (https://genome-cancer.ucsc.edu/proj/site/hgHeatmap/). RNA sequencing data measured by Illumina HiSeq were downloaded whenever available. The colorectal cancer dataset did not contain HiSeq data and gene expression data measured by AgilentG4502_A were downloaded for analysis instead. For the comparison of methylation and gene expression differences between normal and tumor samples, metastatic samples were filtered out using provided annotation data. For the analysis of the breast cancer dataset by subtypes, normal and metastatic samples were removed. Tumors with PAM50 classification information in the TCGA clinical table were used for the analysis. In Figures 4 and 5B the R statistical program [34] was used for statistical analysis and plots were generated using the ggplot2 package [35]. P-values between groups were calculated using Welch's corrected t-test unless otherwise mentioned. Multiple test comparisons were corrected using Bonferroni correction.

## Supporting Information

Figure S1 **Methylation fold difference of 124 Illumina probes as compared breast to colon, endometrial and lung epithelial tissues.**
(PDF)

Table S1 **List of Illumina probes and corresponding genes that were differentially methylated in breast epithelium as compared to colon, lung and endometrial epithelium: Illumina methylation array analysis.**
(XLSX)

Table S2 A. DAVID results: functional classification of 110 genes that had unique methylation in breast epithelium. B. Detailed description of members in the DNA binding and transcription regulators subgroup.
(DOCX)

Table S3 **Genes differentially methylated in normal breast, DNA binding and transcription regulator subgroup: A comparison with methylation in the respective carcinomas, TCGA analysis.**
(DOCX)

Table S4 **TRIM29 CpG island genomic sequence, TRIM29 Illumina probes details and primer sequences for Q-MSP and RT-PCR.**
(PDF)

Table S5 **Comparisons of TRIM29 promoter methylation and gene expression for various normal tissues and respective carcinomas in TCGA.**
(PDF)
