# Supplementary material for: Tissue Specific DNA Methylation in Normal Human Breast Epithelium and in Breast Cancer
Source: PLoS One. 2014 Mar 20;9(3):e91805. doi: 10.1371/journal.pone.0091805 (PMC3961270; doi:10.1371/journal.pone.0091805)

Figure S1. Methylation fold difference of 124 Illumina probes as compared breast to colon, endometrial and lung epithelial tissues.

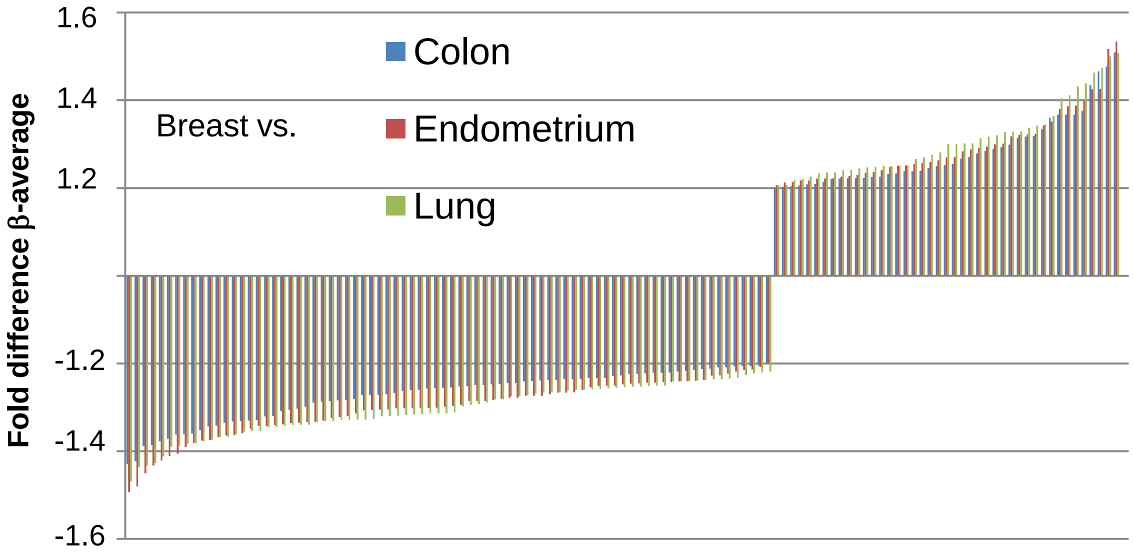

Supplement: Figure S1 — Methylation fold difference of 124 Illumina probes as compared breast to colon, endometrial and lung epithelial tissues. (PDF) [file pone.0091805.s001.pdf]
