# Supplementary material for: Tissue Specific DNA Methylation in Normal Human Breast Epithelium and in Breast Cancer
Source: PLoS One. 2014 Mar 20;9(3):e91805. doi: 10.1371/journal.pone.0091805 (PMC3961270; doi:10.1371/journal.pone.0091805)
Supplement: Table S2 — A. DAVID results: functional classification of 110 genes that had unique methylation in breast epithelium. B. Detailed description of members in the DNA binding and transcription regulators subgroup. (DOCX) [file pone.0091805.s003.docx]

| **Table S2.**   1. **DAVID results** | |
| --- | --- |
| Functional classification of 110 genes that had unique methylation in breast epithelium. | |
|  | |
| **Gene Group 1 - Secreted, extracellular proteins, Enrichment Score: 2.2** | |
| IL20 | interleukin 20 |
| OBP2B | odorant binding protein 2B |
| IL17B | interleukin 17B |
| CDSN | corneodesmosin |
| MUC15 | mucin 15, cell surface associated |
| WFDC6 | WAP four-disulfide core domain 6; serine peptidase inhibitor-like |
| SMR3B | submaxillary gland androgen regulated protein 3B |
| TREM2 | triggering receptor expressed on myeloid cells 2 |
| ADAMTS4 | ADAM metallopeptidase with thrombospondin type 1 motif, 4 |
| PSORS1C2 | psoriasis susceptibility 1 candidate 2 |
| IL17RC | interleukin 17 receptor C |
| DAND5 | DAN domain family, member 5 |
| AMY2A | amylase, alpha 2A (pancreatic) |
| ARSE | arylsulfatase E (chondrodysplasia punctata 1) |
| MIA | melanoma inhibitory activity |
| IL22RA2 | interleukin 22 receptor, alpha 2 |
| CDCP2 | CUB domain containing protein 2 |
| NID1 | nidogen 1 |
|  |  |
| **Gene Group 2 - Calcium homeostasis, Enrichment Score: 1.4** | |
| PROK2 | prokineticin 2 |
| SAA1 | serum amyloid A1 |
| SAA2 | serum amyloid A2 |
| CCKAR | cholecystokinin A receptor |
|  |  |
| **Gene Group 3 - Transcription regulation, Enrichment Score: 1.3** | |
| LYL1 | lymphoblastic leukemia derived sequence 1 |
| ST18 | suppression of tumorigenicity 18 (breast carcinoma) (zinc finger protein) |
| ALX4 | ALX homeobox 4 |
| SOX10 | SRY (sex determining region Y)-box 10 |
| SREBF1 | sterol regulatory element binding transcription factor 1 |
| MGMT | O-6-methylguanine-DNA methyltransferase |
| TP73 | tumor protein p73 |
| FEV | FEV (ETS oncogene family) |
| PAX9 | paired box 9 |
| TRIM29 | tripartite motif-containing 29 |
| HOXA11 | homeobox A11 |
| NEUROG1 | neurogenin 1 |
| ZNF436 | zinc finger protein 436 |
| FHL2 | four and a half LIM domains 2 |
| HLF | hepatic leukemia factor |
| GATA5 | GATA binding protein 5 |
|  |  |
| **Gene Group 4 - Ectoderm and epithelial differentiation, Enrichment Score: 0.9** | |
| CDSN | corneodesmosin |
| LCE1B | late cornified envelope 1B |
| KRT5 | keratin 5 |
| CNFN | cornifelin |
|  |  |
| **Gene Group 5 - transmembrane proteins, Enrichment Score: 0.8** | |
|  |  |
| GPR124 | G protein-coupled receptor 124 |
| SLC26A8 | solute carrier family 26, member 8 |
| CLDN6 | claudin 6; similar to claudin 6 |
| TMEM40 | transmembrane protein 40 |
| TNFSF4 | tumor necrosis factor (ligand) superfamily, member 4 |
| FSHR | follicle stimulating hormone receptor |
| IFNGR2 | interferon gamma receptor 2 (interferon gamma transducer 1) |
| PEX11G | peroxisomal biogenesis factor 11 gamma |
| GPR31 | G protein-coupled receptor 31 |
| MUC15 | mucin 15, cell surface associated |
| CCKAR | cholecystokinin A receptor |
| SLC17A8 | solute carrier family 17 |
| CACNA1G | calcium channel, voltage-dependent, T type, alpha 1G subunit |
| CHRM5 | cholinergic receptor, muscarinic 5 |
| STEAP4 | STEAP family member 4 |
| TMPRSS3 | transmembrane protease, serine 3 |
| TREM2 | triggering receptor expressed on myeloid cells 2 |
| CLDN8 | claudin 8 |
| MT1H | metallothionein 1H |
| FFAR2 | free fatty acid receptor 2 |
| KCTD4 | potassium channel tetramerisation domain containing 4 |
| IL17RC | interleukin 17 receptor C |
| DCST2 | DC-STAMP domain containing 2 |
| ACCN4 | amiloride-sensitive cation channel 4, pituitary |
| MEGF11 | multiple EGF-like-domains 11 |
| ZP4 | zona pellucida glycoprotein 4 |
| SCARA5 | scavenger receptor class A, member 5 (putative) |
| NFAM1 | NFAT activating protein with ITAM motif 1 |
|  |  |
| **Gene Group 6 – Cytoskeleton, Enrichment Score: 0.5** | |
|  |  |
| KRT5 | keratin 5 |
| GFAP | glial fibrillary acidic protein |
| TPM4 | tropomyosin 4 |
| FSD1 | fibronectin type III and SPRY domain containing 1 |
|  |  |
|  |  |
|  |  |
|  |  |

| 1. **Detailed description of members in the DNA binding and transcription regulators subgroup.** | | |
| --- | --- | --- |
| **Gene Symbol** | **Gene**  **Full Name** | **Relevance to differentiation and cancer** |
| ALX4 | Aristaless-like homeobox-4 | ALX4 encodes a paired-like homeodomain transcription factor expressed in the mesenchyme of developing bones, limbs, hair, teeth, and mammary tissue. Mutations or deletions in this gene cause known developmental abnormalities in humans. It was found frequently methylated in colon adenomas, primary and metastatic colorectal cancers, adenocarcinomas of the gastrointestinal tract and in sera of patients [1]. Within a panel of 5 genes, methylation in the plasma was associated with lymph node metastasis in gastric cancer [2]. Also found a putative tumor suppressor with epigenetic silencing in lung carcinogenesis [3]. ALX4 was expressed in both stromal and a luminal epithelial cell in the human breast, and loss of ALX4 expression was found in breast cancers [4]. |
| GATA5 | GATA binding protein 5 | GATA factors are zinc finger DNA binding proteins that control the development of diverse tissues by activating or repressing transcription. GATA factors thus coordinate cellular maturation with proliferation arrest and cell survival, and are involved in many types of cancers [5]. GATA5 hypermethylation was associated with renal cancer metastasis and progression-free survival [6], lung cancer risk [7], gastric carcinoma [8], colon carcinoma [9], pancreatic cancer [10] and eosophagal cancer [11]. In mammary cells, GATA5 could activate the progesterone receptor gene through a mutant variant associated with breast cancer risk [12]. |
| MGMT | O6-methylguanine-DNA methyltransferase | MGMT is a DNA repair enzyme that repairs DNA damage resulting from mutagens and carcinogens. MGMT expression varies greatly in normal tissues, has a developmental role in the brain and in some cases has been related to cancer predisposition. MGMT silencing in various types of tumors, is mainly regulated epigenetically by promoter CpG methylation and in brain tumors this correlates with a better therapeutic response [13]. In breast cancer, MGMT methylation increased with advanced stages [14] and in triple negative subtype [15]. However in ER plus tumors elevated MGMT levels correlated with Tamoxifen resistance [16]. |
| SOX10 | SRY (sex determining region Y)-box 10 | SOX10 is a member of the SOX (SRY-related HMG-box) family of transcription factors involved in the regulation of embryonic development and mediates the survival and differentiation of neural crest cells into glial cells and melanocytes [17]. SOX10 labeling by immunohistochemistry is used clinically primarily to support the diagnosis of melanoma [18]. It is expressed in benign breast myoepithelial cells and preferentially in basal-like, triple negative breast tumors [19]. |
| ST18 | Suppression of tumorigenicity 18 | The ST18 gene encodes a zinc-finger DNA-binding protein. Its homolog in mice Myt/ NZF-3 was shown to play a crucial role together with Neurog1 in neuronal differentiation [20]. It is expressed in a number of normal tissues including mammary epithelial cells although the level of expression is quite low. In breast tumors, ST18 mRNA is significantly downregulated in correlation with hypermethylation of the gene promoter. It was functionally tumor suppressive in cellular and mouse models of breast cancer [21]. |
| TP73 | Tumor suppressor p73 | TP73 is a member of the TP53 family, whose deregulated expression has been reported in a wide variety of cancers and linked to patients' outcome. It encodes a number of isoforms (TAp73 and ΔTAp73) with opposing functions (tumor suppressor or oncogenic) which also cross-talk with other members of the family (TP53 and TP63) [22]. P73 methylation was more frequent in triple-negative breast tumors [15]. |
| TRIM29 (ATDC) | TRIpartite Motif–containing 29 (ataxia telangiectasia group D–complementing). | This gene belongs to the TRIM protein family although it lacks the RING finger domain [23]. It was previously reported that TRIM29 promoted cell proliferation through interaction and suppression of p53 transcriptional activity [24]. Furthermore, over-expression of TRIM29 was observed in many human cancers [23] including pancreatic cancer, where it enhanced tumor growth through stabilization of beta catenin [25]. In contrast, reduced expression of TRIM29 was noted in breast and prostate cancer [26,27] and evidence for growth inhibition and tumor suppression was demonstrated in breast non-malignant and malignant cell lines [28]. |
| SREB1 | Sterol regulatory element binding protein 1 | Sterol regulatory element binding proteins (SREBPs) are a family of transcription factors that regulate genes involved in lipid metabolism, including FAS. SREBP1 was shown to be involved in regulation of lipid biosynthesis in the mammary gland during differentiation [29]. It was shown to play a role in breast cancer [30] ovarian cancer [31], endometrial cancer [32] and other types of cancers. |
| NEUROG1 | Neurogenin1 | The basic helix–loop–helix (bHLH) transcription factors of the Neurogenin (Ngn) family are positive regulators of neurogenesis and inhibitors of gliogenesis [33]. In human cancer, NEUROG1 inhibition and promoter methylation was found in colorectal cancer [34]. |

References

1. Ebert MP, Model F, Mooney S, Hale K, Lograsso J, et al. (2006) Aristaless-like homeobox-4 gene methylation is a potential marker for colorectal adenocarcinomas. Gastroenterology. 131(5): 1418-1430 Epub 2006 Aug 1418.

2. Chen HY, Zhu BH, Zhang CH, Yang DJ, Peng JJ, et al. (2012) High CpG island methylator phenotype is associated with lymph node metastasis and prognosis in gastric cancer. Cancer Sci 103(1): 73-79.

3. Liu WB, Han F, Du XH, Jiang X, Li YH, et al. (2014) Epigenetic silencing of Aristaless-like homeobox-4, a potential tumor suppressor gene associated with lung cancer. Int J Cancer 134(6): 1311-1322.

4. Chang H, Mohabir N, Done S, Hamel PA (2009) Loss of ALX4 expression in epithelial cells and adjacent stromal cells in breast cancer. J Clin Pathol. 62(10): 908-914 doi: 910 1136/jcp 2009 067298.

5. Zheng R, Blobel GA (2010) GATA Transcription Factors and Cancer. Genes Cancer 1(12): 1178-1188.

6. Peters I, Eggers H, Atschekzei F, Hennenlotter J, Waalkes S, et al. (2012) GATA5 CpG island methylation in renal cell cancer: a potential biomarker for metastasis and disease progression. BJU Int 110(2 Pt 2): 30.

7. Leng S, Do K, Yingling CM, Picchi MA, Wolf HJ, et al. (2012) Defining a gene promoter methylation signature in sputum for lung cancer risk assessment. Clin Cancer Res 18(12): 3387-3395.

8. Wen XZ, Akiyama Y, Pan KF, Liu ZJ, Lu ZM, et al. (2010) Methylation of GATA-4 and GATA-5 and development of sporadic gastric carcinomas. World J Gastroenterol 16(10): 1201-1208.

9. Hellebrekers DM, Lentjes MH, van den Bosch SM, Melotte V, Wouters KA, et al. (2009) GATA4 and GATA5 are potential tumor suppressors and biomarkers in colorectal cancer. Clin Cancer Res. 15(12): 3990-3997 doi: 3910 1158/1078-0432 CCR-3909-0055 Epub 2009 Jun 3999.

10. Fu B, Guo M, Wang S, Campagna D, Luo M, et al. (2007) Evaluation of GATA-4 and GATA-5 methylation profiles in human pancreatic cancers indicate promoter methylation patterns distinct from other human tumor types. Cancer Biol Ther. 6(10): 1546-1552 Epub 2007 Jul 1547.

11. Guo M, House MG, Akiyama Y, Qi Y, Capagna D, et al. (2006) Hypermethylation of the GATA gene family in esophageal cancer. Int J Cancer. 119(9): 2078-2083.

12. Huggins GS, Wong JY, Hankinson SE, De Vivo I (2006) GATA5 activation of the progesterone receptor gene promoter in breast cancer cells is influenced by the +331G/A polymorphism. Cancer Res. 66(3): 1384-1390.

13. Christmann M, Verbeek B, Roos WP, Kaina B (2011) O(6)-Methylguanine-DNA methyltransferase (MGMT) in normal tissues and tumors: enzyme activity, promoter methylation and immunohistochemistry. Biochim Biophys Acta 2011(2): 179-190.

14. Klajic J, Fleischer T, Dejeux E, Edvardsen H, Warnberg F, et al. (2013) Quantitative DNA methylation analyses reveal stage dependent DNA methylation and association to clinico-pathological factors in breast tumors. BMC Cancer 13(456): 1471-2407.

15. Branham MT, Marzese DM, Laurito SR, Gago FE, Orozco JI, et al. (2012) Methylation profile of triple-negative breast carcinomas. Oncogenesis 2012(2): 17.

16. Bobustuc GC, Smith JS, Maddipatla S, Jeudy S, Limaye A, et al. (2012) MGMT inhibition restores ERalpha functional sensitivity to antiestrogen therapy. Mol 18:913-29.(doi): 10 2119/molmed 2012 00010.

17. Mollaaghababa R, Pavan WJ (2003) The importance of having your SOX on: role of SOX10 in the development of neural crest-derived melanocytes and glia. Oncogene. 22(20): 3024-3034.

18. Shin J, Vincent JG, Cuda JD, Xu H, Kang S, et al. (2012) Sox10 is expressed in primary melanocytic neoplasms of various histologies but not in fibrohistiocytic proliferations and histiocytoses. J Am Acad Dermatol 67(4): 717-726.

19. Cimino-Mathews A, Subhawong AP, Elwood H, Warzecha HN, Sharma R, et al. (2013) Neural crest transcription factor Sox10 is preferentially expressed in triple-negative and metaplastic breast carcinomas. Hum Pathol 44(6): 959-965.

20. Kameyama T, Matsushita F, Kadokawa Y, Marunouchi T (2011) Myt/NZF family transcription factors regulate neuronal differentiation of P19 cells. Neurosci Lett 497(2): 74-79.

21. Jandrig B, Seitz S, Hinzmann B, Arnold W, Micheel B, et al. (2004) ST18 is a breast cancer tumor suppressor gene at human chromosome 8q11.2. Oncogene. 23(57): 9295-9302.

22. Soldevilla B, Millan CS, Bonilla F, Dominguez G (2013) The TP73 complex network: ready for clinical translation in cancer? Genes Chromosomes Cancer 52(11): 989-1006.

23. Hatakeyama S (2011) TRIM proteins and cancer. Nat Rev Cancer 11(11): 792-804.

24. Yuan Z, Villagra A, Peng L, Coppola D, Glozak M, et al. (2010) The ATDC (TRIM29) protein binds p53 and antagonizes p53-mediated functions. Mol Cell Biol 30(12): 3004-3015.

25. Wang L, Heidt DG, Lee CJ, Yang H, Logsdon CD, et al. (2009) Oncogenic function of ATDC in pancreatic cancer through Wnt pathway activation and beta-catenin stabilization. Cancer Cell. 15(3): 207-219 doi: 210 1016/j ccr 2009 1001 1018.

26. Ernst T, Hergenhahn M, Kenzelmann M, Cohen CD, Bonrouhi M, et al. (2002) Decrease and gain of gene expression are equally discriminatory markers for prostate carcinoma: a gene expression analysis on total and microdissected prostate tissue. Am J Pathol. 160(6): 2169-2180.

27. Nacht M, Ferguson AT, Zhang W, Petroziello JM, Cook BP, et al. (1999) Combining serial analysis of gene expression and array technologies to identify genes differentially expressed in breast cancer. Cancer Res. 59(21): 5464-5470.

28. Liu J, Welm B, Boucher KM, Ebbert MT, Bernard PS (2012) TRIM29 functions as a tumor suppressor in nontumorigenic breast cells and invasive ER+ breast cancer. Am J Pathol 180(2): 839-847.

29. Anderson SM, Rudolph MC, McManaman JL, Neville MC (2007) Key stages in mammary gland development. Secretory activation in the mammary gland: it's not just about milk protein synthesis! Breast Cancer Res. 9(1): 204.

30. Pandey PR, Xing F, Sharma S, Watabe M, Pai SK, et al. (2013) Elevated lipogenesis in epithelial stem-like cell confers survival advantage in ductal carcinoma in situ of breast cancer. Oncogene 32(42): 5111-5122.

31. Nie LY, Lu QT, Li WH, Yang N, Dongol S, et al. (2013) Sterol regulatory element-binding protein 1 is required for ovarian tumor growth. Oncol Rep 30(3): 1346-1354.

32. Li W, Tai Y, Zhou J, Gu W, Bai Z, et al. (2012) Repression of endometrial tumor growth by targeting SREBP1 and lipogenesis. Cell 11(12): 2348-2358 doi: 2310 4161/cc 20811 Epub 22012 Jun 20815.

33. Sun Y, Nadal-Vicens M, Misono S, Lin MZ, Zubiaga A, et al. (2001) Neurogenin promotes neurogenesis and inhibits glial differentiation by independent mechanisms. Cell. 104(3): 365-376.

34. Herbst A, Rahmig K, Stieber P, Philipp A, Jung A, et al. (2011) Methylation of NEUROG1 in serum is a sensitive marker for the detection of early colorectal cancer. Am J Gastroenterol 106(6): 1110-1118.
