# Supplementary material for: Tissue Specific DNA Methylation in Normal Human Breast Epithelium and in Breast Cancer
Source: PLoS One. 2014 Mar 20;9(3):e91805. doi: 10.1371/journal.pone.0091805 (PMC3961270; doi:10.1371/journal.pone.0091805)
Supplement: Table S3 — Genes differentially methylated in normal breast, DNA binding and transcription regulator subgroup: A comparison with methylation in the respective carcinomas, TCGA analysis. (DOCX) [file pone.0091805.s004.docx]

**Table S3**

Genes differentially methylated in normal breast, DNA binding and transcription regulator subgroup: A comparison with methylation in the respective carcinomas–TCGA analysis.

15 DNA binding and transcription regulators with breast specific methylation in normal tissues were analyzed for differential methylation in normal versus tumor samples. TCGA Illumina methylation 450 data sets of breast (BRCA), colon (COAD), lung (LUSC) and endometrial (UCEC) cancers were analyzed in the UCSF genome browser. Normal samples were selected as "normal solid tissues" and cancers as "primary tumors" and compared by student t-test with Bonferroni correction. Hyper-methylation in the tumors. Hypo-methylation in the tumors. P-values (-log_10_) are given for each comparison. "ns" not significant, p>0.05. The Illumina probe ID refers to the probe that was found differentially methylated in the breast (Table S1). For some genes, more than one probe was included in the analysis.. Sample numbers: (N=normal, T=tumors) breast: N=79 T=497, colon: N=16, T=150, lung: N=42, T=226, endometrium: N=42, T=383. Additional information for the probe genomic site is supplied.
