# Supplementary material for: Tissue Specific DNA Methylation in Normal Human Breast Epithelium and in Breast Cancer
Source: PLoS One. 2014 Mar 20;9(3):e91805. doi: 10.1371/journal.pone.0091805 (PMC3961270; doi:10.1371/journal.pone.0091805)
Supplement: Table S4 — TRIM29 CpG island genomic sequence, TRIM29 Illumina probes details and primer sequences for Q-MSP and RT-PCR. (PDF) [file pone.0091805.s005.pdf]

Table S4

TRIM29 CpG island genomic sequence, Illumina probes details and primers for Q-MSP and RT-PCR.

(A)

TGGAGGGGCAGGAGGGGCCCCAGCACTTACCTCCTTCT**CG**GCCTTGGCCTCCTCCACTGTCA**CG**GTGCTATGATTCTTGTGCTCCTGGAACATGCAAAGG
TAGCAGATGCAGGTCTGGT**CG**GTCTGGCAGAAGAGCTCCAT**CG**TCTTGCCATGCA**CG**GGACACTTG**CG**GGCCTCAAAGTCC**CG**GATGGGCT**CG**AGCAGCT
GGTGGTCT**CG**GAAGG**CG**G**CG**CCCTCCAGGTGGGGCTTGAGATGCAGCT**CG**CAGAAGGAGGCCTGGCACACCAGGCAGGACTTGAC**CG**CCTTCTGCTTGTT
GC**CG**ATGCAGGAGT**CG**CACAGCACCTCCT**CG**GAGC**CG**GACTTGGAC**CG**TGAAAAAAGGCC**CG**TGT**CG**GCC**CG**GGGGTAGCTGTTC**CG**C**CG**GGTCTCCC**CG**
GGCTCCATGATGGACAC**CG**TGGGCTTC**CG**GGACTC**CG**AGAAAATGGACTTG**CG**CAGCT**CG**CCCTTTT**CG**GCAAAGGTAA**CG**GGTGGCTTCTTGGCAGCCC
CC**AGCTGGAGCCCTGCGTACGGCG**ACCTCTTGCCTTCCATAGAGTCCATGCTGAAGTAGTTGGAGTTCTTGT**CGTCCCCGGACTCGACAAACT**GGATGAT
GGGT**CG**C**CG**CCACTCATTGCC**CGCG**AACAGGG**CG**CTCCTACCTTCCCCT

TRIM29 CpG island genomic sequence 648 bases, chr11:119513116-119513764 (located 300 b away from TSS at chr11:119514073). From NCBI Reference Sequence: NC_000011.9, Homo sapiens chromosome 11, GRCh37.p2 primary reference assembly. The Illumina infinium methylation array 450K probes are marked in 5 colors as listed in B.

(B)

IlmnID	SourceSeq	Chr	Coordinate_36
cg20655548	GATTCTTGTGCTCCTGGAACATGCAAAGGTAGCAGATGCAGGTCTGGTCG	11	119513235
cg12201660	ACACCAGGCAGGACTTGACCGCCTTCTGCTTGTTGCCGATGCAGGAGTCG	11	119513430
cg17971587	AAAAGGGCGAGCTGCGCAAGTCCATTTTCTCGGAGTCCCGGAAGCCCACG	11	119513533
cg13285004	CGGGGACGACAAGAACTCCAACTACTTCAGCATGGACTCTATGGAAGGCA	11	119513694
cg13625403	TAGTTGGAGTTCTTGTCGTCCCCGGACTCGACAAACTGGATGATGGGTCG	11	119513720

TRIM29 Illumina probes: detailed list of the 5 probes that were analyzed in ENCODE and in TCGA

(C)

Tissue type	Ave.beta	Tissue type	Ave.beta
negative control_1	0.020	colon_1	0.935
negative control_2	0.062	colon_2	0.911
positive control_1	0.911	lung_1	0.957
positive control_2	0.861	lung_2	0.944
breast_1	0.488	endometrium_1	0.968
breast_2	0.501	endometrium_2	0.955
breast_3	0.518	WBC_1	0.956
breast_4	0.651	WBC_2	0.955
breast_5	0.545	WBC_3	0.959
breast_6	0.454		

Illumina Infinium methylation array27K results for TRIM29 probe # cg13625403 – methylation was measured as average beta 0-1.

(D) Primers for TRIM29 Q-MSP

Design was based on the NCBI Reference Sequence: NC_000011.9, Homo sapiens chromosome 11, GRCh37.p2 primary reference assembly. Note that the sequence was adjusted for methylation dependent CT conversion by sodium bi-sulfite.

Unmethylated : **TRIM29 FUM** (forward) TTTAGTTTGT**TG**AGTT**TG**GGGA**T** **TRIM29 RUM** (reverse) CAACTAAAACCCTA**CA**TA**CA**A**CA**

Methylated: **TRIM 29 FM** (forward) AGTTTGT**CG**AGTT**CG**GGGA**C** **TRIM 29 RM** (reverse) ACTAAAACCCTA**CG**TA**CG**A**CG**

(E) Primers for gene expression

Design was based on the NCBI Reference Sequence: NM_012101.3 and used NCBI primer blast program.

TRIM29_GEXF (forward) CTGACAATGACCTGCC**CG**T**CG**TCCA

TRIM29_GEXR (reverse) CCTTGGGGCTTTGGCTC**CG**CAT
