# Supplementary material for: Tissue Specific DNA Methylation in Normal Human Breast Epithelium and in Breast Cancer
Source: PLoS One. 2014 Mar 20;9(3):e91805. doi: 10.1371/journal.pone.0091805 (PMC3961270; doi:10.1371/journal.pone.0091805)
Supplement: Table S5 — Comparisons of TRIM29 promoter methylation and gene expression for various normal tissues and respective carcinomas in TCGA. (PDF) [file pone.0091805.s006.pdf]

Table S5.

Comparisons of TRIM29 promoter methylation and gene expression for various normal tissues and respective carcinomas in TCGA

(A)

	BLCA	BRCA	COAD	LUSC	PAAD	PRAD	READ
BRCA	1	-	-	-	-	-	-
COAD	0.04378801	2.09E-05	-	-	-	-	-
LUSC	0.09690264	2.20E-07	1	-	-	-	-
PAAD	1	0.07006369	1	1	-	-	-
PRAD	2.68E-13	2.91E-23	2.74E-24	6.56E-35	2.20E-12	-	-
READ	0.06775645	0.00112829	1	1	1	1.41E-15	-
UCEC	1.07E-06	4.30E-19	1	1.52E-02	1.00E+00	6.82E-48	1

Multiple comparisons of TRIM29 promoter methylation in various normal tissues. TCGA data used as described in methods. Analysis for 5 Illumina probes at the TRIM29 TSS region (probes detailed in supplementary table 3). P-values (average for 5 probes) between groups were calculated by t-test and Bonferroni correction. ▢ $p<0.05$.

(B)

	BLCA	BRCA	COAD	LUSC	PAAD	PRAD	READ
BRCA	1.12E-66	-	-	-	-	-	-
COAD	1.12E-27	0.00161169	-	-	-	-	-
LUSC	0.06378519	8.07E-59	4.23E-19	-	-	-	-
PAAD	1.36E-24	1	0.17668472	4.57E-18	-	-	-
PRAD	3.29E-06	4.57E-33	2.81E-09	0.21634977	1.61E-11	-	-
READ	3.49E-09	2.59E-12	0.01105954	0.00050761	6.08E-06	1	-
UCEC	8.78E-45	0.00112701	1	2.89E-35	0.55122249	1.75E-17	2.98E-05

Multiple Comparisons of TRIM29 promoter methylation in various cancers. TCGA data used as described in methods. Analysis for 5 Illumina probes at the TRIM29 TSS region (probes detailed in supplementary table 3). P-values (average for 5 probes) between groups were calculated by t-test and Bonferroni correction. ▢ $p<0.05$.

(C)

	BLCA	BRCA	LUSC
BRCA	1	-	-
LUSC	0.00069134	9.74E-11	-
PRAD	0.60609728	0.79538649	7.69E-11

Comparisons of TRIM29 gene expression in various normal tissues. TCGA data used as described in methods. P-values were calculated by t-test and Bonferroni correction. ▢ $P<0.05$

(D)

	BLCA	BRCA	LUSC
BRCA	1	-	-
LUSC	0.00069134	9.74E-11	-
PRAD	0.60609728	0.79538649	7.69E-11

Comparisons of TRIM29 gene expression in various cancers. TCGA data used as described in methods. P-values were calculated by t-test and Bonferroni correction. ▢ $P<0.05$

(E) Number of samples in TCGA analysis

methylation	n (normal)	n (cancer)
BRCA	97	497
PRAD	49	176
LUSC	42	226
BLCA	18	153
PAAD	7	49
COAD	16	150
READ	7	95
UCEC	42	383

expression	n (normal)	n (cancer)
BRCA	107	844
PRAD	39	140
LUSC	35	259
BLCA	16	122

For all tables: BRCA=breast carcinoma, PRAD=prostate adenocarcinoma, LUSC=lung squamous cell carcinoma, BLCA=bladder carcinoma, PAAD=pancreatic adenocarcinoma, COAD=colon adenocarcinoma, READ=rectal adenocarcinoma UCEC=uterine-cervix carcinoma. Number of samples in each group is shown in (E).
